# Supplementary material for: Are carbon emissions trading and green financial instruments synergistic? -Comprehensive quantitative research based on content analysis
Source: PLoS One. 2024 Mar 7;19(3):e0298601. doi: 10.1371/journal.pone.0298601 (PMC10919601; doi:10.1371/journal.pone.0298601)
Supplement: S1 Data — (ZIP) [file pone.0298601.s001.zip › Data/地级市Z_F工作报告.zip!/╕ú╜¿/╞╬╠∩╩╨/└┤╫╘╣1⁄2╓┌║┼í╛┬φ┐╦╩2╛▌═°í┐_2019.docx]

各位代表：

　　现在，我代表莆田市人民政府，向大会报告政府工作，请予审议，并请市政协委员提出意见。

　　一、2018年工作回顾

　　过去一年，我们在以习近平同志为核心的党中央坚强领导下，坚持以习近平新时代中国特色社会主义思想为指导，认真贯彻党的十九大和十九届二中、三中全会精神，认真贯彻习近平总书记对福建工作的重要指示精神和治理木兰溪的重要理念，认真贯彻党中央国务院、省委省政府和市委决策部署，坚持新发展理念，坚持稳中求进工作总基调，以供给侧结构性改革为主线，打好“三大攻坚战”，全力推动高质量发展落实赶超，新动能加快培育，城市加快更新升级，经济社会保持健康平稳发展，荣获“国家森林城市”，创成“全国水生态文明城市”，较好完成了市七届人大二次会议确定的目标任务。

　　初步统计，地区生产总值2252亿元、增长8.4%；一般公共预算总收入225.9亿元、增长7%，地方一般公共预算收入141亿元、增长3.4%；固定资产投资增长13%；社会消费品零售总额760亿元、增长9.4%；外贸出口总额220亿元、增长6%；实际利用外资8.66亿元；居民人均可支配收入2.8万元、增长8.7%；城镇登记失业率2.5%；居民消费价格总水平上涨2%。

　　2018年，我们共同经历了一系列激动人心、值得铭记的大事：

　　这一年，中央主要媒体集中报道习近平总书记治理木兰溪重要理念。20年来，历届市委市政府接续奋斗、久久为功，实现了习近平总书记当年提出的“变害为利、造福人民”目标，木兰溪全流域系统治理经验成为建设美丽中国的生动范本，在全国宣传推广，引起全社会热烈反响，我们倍受鼓舞、倍感振奋！

　　这一年，第五届世界佛教论坛在我市成功举办，全市上下勠力同心、众志成城，推动力度之大、速度之快创历届之最，参会的国家和地区数量创历届之最，各方好评、效果之好创历届之最。论坛的成功举办汇聚了积极向上的正能量，提振了干部的精气神，增强了市民的凝聚力，把莆田推向一个新的层次，让城市形象跃升一个新的台阶。

　　这一年，我们牢记习近平总书记关于保护好湄洲岛的嘱托，围绕生态岛、文化岛、旅游岛、智慧岛的发展目标，实行最严格的生态保护制度，在住建部指导下开展城市智慧汽车基础设施及机制建设试点工作，为未来智慧城市建设提供示范。成功举办第三届世界妈祖文化论坛，参与国家和地区更多，内涵更丰富，妈祖文化的影响力越来越大。

　　一年来，我们主要做了以下工作：

　　（一）凝心聚力打好“三大攻坚战”。完善政银企司合作机制，加大不良贷款处置力度，年内不良率从最高的4.25%回落至2.65%；落实房价地价“双控”，房地产市场平稳健康发展；深入开展扫黑除恶专项斗争、安全生产“严整治、促提升”专项行动，社会保持安定稳定；成功处置非洲猪瘟疫情，防控工作有序有效。扎实开展精准脱贫百日攻坚战，建成产业扶贫基地206个，32个贫困村脱贫摘帽，贫困对象的获得感和满意度不断提升。坚决落实中央、省环保督察整改任务，深入开展污染防治攻坚战六大专项行动，整治“散乱污”企业893家，重点企业的废气治理设施全部落实到位；大力开展木兰溪流域水质提升攻坚行动，实行“双河长”制，取缔“反弹复建”畜禽养殖场1034家，综合治理河道773公里，小流域劣Ⅴ类水质提升任务提前完成，劣Ⅴ类水质断面全面消除；建成东圳水库水环境综合治理、木兰溪玉湖段生态提升工程等一批水生态项目。

　　（二）千方百计帮扶实体经济。坚持“两个毫不动摇”，深入开展企业大走访活动，创新分级走访、全程跟踪、问题分办等工作机制，一批企业反映的融资难、招工难等热点问题得到解决。出台稳定工业、金融服务、扶持外贸、促进工业园区和民营经济健康发展等系列措施，全年为企业减负32亿元。提升营商环境，激发市场活力，新增商事主体8.37万户，增长80.3%，“个转企” “下转上”分别超过7300家、1100家；有效投资持续扩大，制造业投资增长13%、技改投资增长40%、民间投资增长30%，新增“五个一批”项目1300个，500个市重点项目和30个美丽莆田重点攻坚项目分别完成投资1150亿元、409亿元。

　　（三）深入推进产业优化升级。坚持新兴产业集聚集约集群发展，华佳彩一期、砷化镓、HDT高效太阳能电池、元生智汇、华锦实业、永荣CPL一期、上海电气等项目实现量产或试投产，创世纪超算中心、三棵树高新材料产业园、手机配件产业园等项目加快建设，合力泰、京东数字经济产业园、杰木芯片等项目签约落地，战略性新兴产业增加值增长18%。坚持两化融合、智能制造，运用新技术、新业态、新模式，改造提升传统产业，鞋服产业产值突破千亿。大力培育高新企业，新认定高新技术企业31家、科技小巨人领军企业11家，高技术产业增加值增长20%以上。落实“五个一”机制，特色小镇和文化名胜区创建工作扎实推进。大力扶持平台经济，推动优势产业集聚升级，平台交易总额突破200亿元。推进全域生态旅游加快发展，菜溪岩获评国家4A级旅游景区，改造升级24家星级酒店，全年接待游客3500万人次、旅游总收入350亿元，分别增长23%、31%。大力实施乡村振兴战略，组建全省首家振兴乡村集团，朗桥、张洋等一批田园综合体建设初见成效。

　　（四）全力提升城乡规划建设管理水平。开展城乡规划建设管理提升年行动。加快一批重点片区建设，建成棚改房6804套，会展中心、规划展示馆、美术馆等一批公共设施投入使用，改造后的南湖公园人气爆棚，“一溪两场”等夜景工程点亮莆田，城市“颜值”明显提升。加快基础设施建设，福厦客专、联十一线、纵三线等重大交通项目加快推进，黄石高速互通、城港大道快速化、动车站前路网等完成提升改造，新改建城乡道路138公里、城镇污水管网98公里，市政供水、燃气管网不断完善。新增公交线路5条，新建城市绿道39公里、人行天桥6座，建成中心城区和湄洲岛的公共自行车系统，骑行量超百万人次，绿色出行成为新风尚。整治城市对外通道和城区主次干道，拆除“两违”202万平方米。开展“厕所革命”，新建和升级改造公厕401座。新改建农村三格化粪池5.8万户，完成251个村庄污水整治。

　　（五）不断激发改革开放活力。各项改革任务取得成效。社会信用体系建设成效显著，在全国262个地市诚信考评中居第19位，在全省首创信访“诚信+”机制。“放管服”改革持续深化，95%以上审批服务事项实现“一趟不用跑”“最多跑一趟”，“多证合一”改革走在全国前列。国企改革持续推进，市属国企总资产达900亿元。扩大对外开放，莆田港东吴港区扩大开放通过国家验收，东南沿海最大的铁矿石码头建成投用；成功举办妈祖巡安菲律宾等活动，加入21世纪海上合作委员会，与英国爱丁堡市缔结友好城市。莆台交流不断深化，开展“台商台胞服务年”活动，出台“35条”惠台政策。坚持协同联动，主动融入闽东北协同发展区建设。

　　（六）加快发展社会民生事业。30项为民办实事完成29项。构建全域就业社保服务体系，城镇新增就业超过1.8万人；实施全民参保计划，养老、医疗保险参保率居全省前列。创办全国首批新工科学院、当年实现招生，莆田学院、湄职院、莆田一中新校区加快建设，湄职院入围省级现代示范校。实施教育强市十大工程，新改扩建幼儿园项目28个、中小学项目53个，新增幼儿园学位7380个、中小学学位13590个。仙游县总医院挂牌成立，市第一医院新门诊和病房大楼全面投用，实施医药卫生人才“三大工程”，新增执业医师717名，推进中医固本三年行动，建成基层“中医馆”23家。建成农村幸福院388所，新增养老床位2311张。文化事业更加繁荣，市博物馆、图书馆、科技馆开馆运行，建成莆阳书房示范点6个，新增省级以上非遗传承人24名，莆仙戏《踏伞行》获省第27届戏剧会演一等奖。开展全民健身运动，成功举办第二届“妈祖杯”海上丝绸之路国际羽毛球挑战赛，组织参加第十六届省运会，获金牌37枚。

　　2018年，我们还持续争创全国双拥模范城，完成新一届村（居）委会换届选举工作，开展了第三次全国国土调查及侨情普查工作。粮食安全、食品药品、档案、民族宗教、工会、妇女儿童、青少年、残疾人、红十字会等工作取得新成效。

　　各位代表，过去一年，我们始终把党的政治建设摆在首位，持续兴起习近平新时代中国特色社会主义思想“大学习”热潮，树牢“四个意识”，坚定“四个自信”，坚决做到“两个维护”，深入推进“两学一做”学习教育常态化制度化，全面落实意识形态工作责任制，切实加强政府自身建设，完成政府机构改革设置。认真落实全面从严治党要求，压紧压实“两个责任”，狠抓中央巡视反馈问题整改，持续落实中央八项规定和实施细则精神，坚决防止“四风”反弹回潮，政府系统党风廉政建设和反腐败工作不断深入。大力弘扬“马上就办、真抓实干”作风，深入推进机关效能建设，落实正向激励和容错纠错机制，政府执行力进一步提升。坚决执行市人大及其常委会各项决议决定，自觉接受人大法律监督、工作监督和政协民主监督，坚持重大事项向市人大报告、向市政协通报，提请市人大常委会审议法规草案3件，研究处理人大常委会审议意见14件，办结率100%，办理人大代表建议182件、政协提案205件，办复率均为100%。强化重大行政决策合法性审查，行政执法和行政复议更加规范，政府信息公开工作不断加强。

　　各位代表，2018年政府工作报告共分解工作任务626项,已完成及基本完成的任务609项、未完成17项，实际完成率97.3%。地区生产总值、一般公共预算总收入、地方一般公共预算收入、固定资产投资增幅没有达到年初预期。对于未完成的任务，我们已责成相关单位作书面解释说明。今后，我们将进一步加大责任落实和工作推进力度，全力以赴完成各项目标任务。

　　各位代表，成绩来之不易，这是在习近平新时代中国特色社会主义思想科学指引下取得的结果，是在省委省政府和市委坚强领导下，在市人大、市政协的监督支持下，全市人民撸起袖子干出来的。在这里，我代表市人民政府，向全市人民，向给予政府工作大力支持的人大代表和政协委员，向各民主党派、各人民团体和社会各界，向驻莆部队、武警官兵，向港澳台同胞、海外乡亲和国际友人，表示衷心的感谢！

　　同时，我们也清醒地认识到存在的不足和问题，主要是：外部环境趋紧，部分企业经营困难增多，外经贸发展难度加大；产业升级仍处于爬坡越坎阶段，新动能未能挑起大梁；城市基础设施还有不少短板；基本公共服务供给仍然不足，群众在上学、看病、养老等方面还有不满意的地方；生态保护和污染防治任务依然艰巨；思想观念还不够解放，传统思维、路径依赖、不担当不作为等问题仍然存在，营商环境亟需改善。对此，我们将切实增强忧患意识，尽心竭力加以解决。

　　二、2019年主要工作

　　今年是新中国成立70周年，是全面建成小康社会关键之年，也是我市实现赶超目标的关键之年。政府工作的总体要求是：以习近平新时代中国特色社会主义思想为指导，全面贯彻党的十九大和十九届二中、三中全会精神，深入贯彻习近平总书记对福建工作的重要指示精神和治理木兰溪的重要理念，统筹推进“五位一体”总体布局和协调推进“四个全面”战略布局，认真落实中央、省委和市委经济工作会议精神，紧紧围绕新福建建设，坚持稳中求进工作总基调，坚持新发展理念，坚持高质量发展落实赶超，坚持以供给侧结构性改革为主线，坚持深化市场化改革、扩大高水平开放，继续打好“三大攻坚战”，加快实体经济创新转型，加快建设现代化经济体系，主动融入闽东北协同发展区，统筹推进稳增长、促改革、调结构、惠民生、防风险工作，进一步稳就业、稳金融、稳外贸、稳外资、稳投资、稳预期，提振市场信心，增强人民获得感、幸福感、安全感，保持经济持续健康发展和社会大局稳定，努力为建设美丽中国提供生动范本，为全面建成小康社会收官打下决定性基础，以优异成绩落实赶超任务、庆祝新中国成立70周年。主要预期目标是:地区生产总值增长9%左右;规模以上工业增加值增长9%；固定资产投资增长13%;社会消费品零售总额增长12%;外贸出口总额持平;实际利用外资增长6.5%；一般公共预算总收入增长8%，其中地方一般公共预算收入增长5%;R&D投入占比1.2%以上;城乡居民人均可支配收入增长9.5%；城镇登记失业率控制在3%以内；居民消费价格总水平涨幅3%左右；完成节能减排降碳任务。

　　实现上述目标，我们要重点做好以下工作:

　　（一）始终坚持把创新作为高质量发展落实赶超的第一动力，大力实施创新驱动发展战略，不断增强经济创新力和竞争力

　　激发企业创新活力。完善促进企业创新的加计扣除等正向激励机制，加大科技成果转化和技改资金支持，落实财政奖励与企业贡献、研发投入等绩效挂钩措施，激励企业技术创新、工艺创新、管理创新、模式创新。建立公共技术研发服务平台，支持企业抱团建立创新联盟，推动企业与科研单位共同组建创新联合体，强化产业核心技术和上下游应用技术攻关，实现科技创新链和产业链精准对接，提升企业创新能力。实施“专精特新”“隐形冠军”企业培育计划和高新技术企业成长计划，新增高新技术企业30家以上、高成长企业30家以上，支持永荣、华峰等建设国家级重点实验室，培育一批示范企业。

　　突出抓好创新载体。将中科院海西研究院莆田中心、中电智能制造研究院、中关村医学工程转化（福建）分中心等打造成高技术成果转移转化基地、产业技术源头创新基地。围绕构建“众创空间-孵化器-加速器”的全链条服务，鼓励盘活老旧厂房、闲置房屋、商业设施等闲置资源，打造一批创新工场、创客空间、大学生创新创业基地，新增市级以上众创空间10家。强化与工信部直属院校、科研机构和国内外其他知名科研院所、智库合作，建设协同创新平台。

　　强化创新人才保障。支持企业与本地院校及国内外知名院校、机构开展全方位、多层次人才培养合作，加快培育一批科技领军人才、青年科技人才和高水平创新团队，壮大面向生产一线的实用人才和专技人才队伍。完善以“用”为导向的人才评价机制、激励机制，让创新人才“名利双收”。举办人才沙龙，推广柔性引才，只求所用、不求所在，让更多海内外人才“智汇莆田”。优化“壶兰计划”，组建人才发展集团、产业人才金融运营公司，加快人才社区、双语学校、国际医院等高端配套建设，让各路英才扎根莆田。

　　（二）深化供给侧结构性改革，推动制造业高质量发展，加快建设现代产业体系

　　推动新兴产业快速发展。组建新兴产业投资控股公司，建立产业培育推进机制，跟踪辨识国际先进前沿技术，编制分行业发展路线图，抢抓大数据、人工智能发展机遇，发展上中下游密切衔接、配套完善的新兴产业体系。电子信息产业，紧盯5G时代，推进“强屏补芯”，依托华佳彩、砷化镓、HDT高效太阳能电池、元生智汇、合力泰等龙头，主动对接台湾地区和京沪深的电子信息产业，重点发展集成电路、新型显示面板等产品，推动手机等全产业链集聚发展。化工（纺织化纤）新材料产业，依托永荣CPL、PA6、华峰、三棵树、佳通轮胎、赛得利等龙头，加快关联企业上下游产品协作配套，推动尼龙、涂料和纺织新材料全产业链发展，打造精细化工终端产业集群。医疗健康产业，围绕妈祖国际健康城开业运营目标，建成投用6家专科医院和医技共享中心；加快秀屿和北岸医药器械制造产业园建设，推动威高、霖扬生技等一批项目入驻；联合国药集团，举办面向全国民营医院的大型药品药械展会；加快设立创投基金、健康产业基金、莆籍民营医院并购基金、国际商业保险公司，推动医疗康养、研发转化、教育培训协同发展，延伸健康全产业链，争取获批两岸医疗健康产业合作试验区。高端装备制造业，以云度汽车为龙头，加强与中国一汽等国内外汽车大品牌、大公司的战略合作，开展关键零部件、核心技术的产业链招商，加快建设新能源汽车产业园，推动创建汽车小镇；支持上海电气、海山机械提升产能、拓展市场，加大节能环保装备研发，发展高端数控机床、工业机器人等智能装备。

　　加快传统产业优化升级。加强技术革新、联盟创新，运用智能化、大数据和“互联网+”手段，为传统产业赋能升级。鞋服产业，发挥制造端优势，推广智能化改造，加强供应链整合、新材料应用、技术需求库建设，鼓励发展个性化定制、众筹众包等新模式，建设个性化定制展演中心，打造“中国军鞋莆田造”区域品牌，做强产业生态圈，让更多高质量的“莆田好鞋”走向世界。工艺美术产业，依托仙作供应链、大工美之家等公共服务平台，嫁接工信部工业文化发展中心、华润中艺、中央美术学院等高端资源，投用国家级质量检测鉴定中心、研发设计院、京东数字经济产业园（一期），策划对接国内顶级展览展示、拍卖、设计活动，提升红木家具、金银珠宝的品牌效应。食品加工产业，以雪津、和润为龙头，瞄准千亿产业集群，延伸粮油食品精深加工、啤酒饮料生产、麦芽、麦汁、包装等全产业链，实现跨越发展。建筑产业，鼓励企业开展资质升级和专业拓展，建设生产基地和向外拓展市场；大力引进施工总承包特级、一级资质企业，筹建建筑劳务、设备租赁、地材集中供应平台，发展建筑业总部经济；推广智能和装配式建筑，推行工程总承包，发展工程建设新业态新模式。

　　推动现代服务业提速提质。推动先进制造业与现代服务业深度融合，发展个性化定制、主辅分离、整体方案解决等服务型制造，培育服务型制造示范企业。实施新一轮莆商回归工程，积极引进一批龙头企业在莆设立区域总部、研发总部、营销总部。加快扶持和引进各类金融机构，大力支持企业改制上市，扩大直接融资规模，强化保险保障和增信功能，发展普惠金融、绿色金融、科技金融，建立产值、税收、就业与融资联动激励机制，实现产业与金融发展的良性互动。发展电商服务业和应用型信息服务业，培育定制消费、体验消费、绿色消费、共享消费等消费新热点，促进服务消费加快提质扩容。实施全域旅游行动提升计划，进一步拓展第五届世界佛教论坛“后效应”，策划一批具有浓郁特色的标志性旅游项目，加快一批重点景区提档升级，促进文化和旅游融合发展。创新“物流+互联网+金融”模式，培植壮大物流龙头企业，打造多式联运物流示范基地。

　　（三）聚焦平台建设，促进高端要素集聚和产业跨界融合，为高质量发展落实赶超积蓄新动能

　　大力发展平台经济。立足线下优势资源，运用互联网思维、大数据技术、资本纽带和品牌植入，提升平台定位，创新平台模式。加大政策扶持，壮大供需两端，提升价值链，贯通供应链，盘活金融链，加速平台交易增值裂变。重点做大做强珠宝云、药械网、物泊物流无车承运、智慧能源、众协联等一批平台，交易总额突破1000亿元，打造全省平台经济集聚示范区，带动线下形成采购中心、销售中心、财务中心和高附加值产业集聚。加强会展中心招商引展工作，引入市场化机制，搭好艺博会、红博会、啤酒节、珠宝展等平台，大力发展会展经济。

　　推动特色小镇高质量发展。立足产业优势，因地制宜，走“产、城、人、文”融合的内涵式发展、市场化运作路子，不搞大拆大建，不搞盲目举债，不搞“自娱自乐”，充分调动各方面积极性，聚焦主业，构建生态，创新模式，彰显特色。推动妈祖国际健康城、金银谷等特色小镇对标国际、嫁接高端、延伸产业链，发展新业态；推动冰雪、啤酒等特色小镇强化招商引资，引进社会资本和专业运营团队，实现良性运营和可持续发展；推动仙作、鞋艺、艺雕等特色小镇盘活资源，搭建平台，集聚人才；推动南少林、九鲤湖等名胜区提升配套设施，丰富文化内涵，打响品牌。

　　推进园区高质量发展。落实促进园区高质量发展三年行动计划，编制园区整合提升规划，实施六大提升专项行动，开展“园中村、村中园”整治，完善基础设施，提升公共配套。推行“管委会+公司”治理模式，成立园区运营公司，设立园区产业发展基金，赋予园区更多财权、事权。开展园区项目评审，处置一批僵尸企业，通过收储闲置土地、“腾笼换鸟”、代建厂房、弹性供地等，提高园区亩产综合效益，争创制造业高质量发展示范区。

　　（四）坚持高起点高品质规划建设，提升城市能级和核心竞争力，精心打造美丽宜居现代城市

　　全力保护好湄洲岛。牢记习近平总书记关于保护好湄洲岛的嘱托，编制湄洲岛“五位一体”综合发展战略规划，严格保护好妈祖文化遗产、风景名胜资源、自然生态环境，开展岸线修复和沙滩保洁，严厉打击“两违”和非法采砂，提高管理水平。加快“城市智慧汽车基础设施及机制建设试点”工作，推进全岛电动化出行、零排放清洁能源建设，打造全域智能网联智慧岛。启动湄洲岛旅游第二通道建设，推进“海上丝绸之路·中国史迹”申报世界遗产，提升第四届世界妈祖文化论坛办会水平，不断扩大妈祖文化品牌影响力。

　　优化城市功能布局。深化“多规合一”，探索建立统一的空间规划体系，构建“山水城田林海”相得益彰的城市格局。推动城市“东拓南进西联”，加快推进玉湖、大学城、高铁、木兰等重点片区开发建设。深入开展“城市双修”，改造提升坂头东等一批棚户区、老旧小区。强化科学谋划，创新运作机制，统筹资金平衡，做到建设一片、繁荣一片、带动一片，提升城市的科教、研发、文创、总部、会展等现代服务功能。

　　加快基础设施建设。构建综合交通发展新格局，加快筹建妈祖机场，争取开工建设沈海高速扩容二期，建成联十一线涵江及仙游段、纵三线。加快推进木兰大道、文献路东拓、八二一街南伸、滨溪北路等主次干道建设，打通涵港大道、南园路延伸等“断头路”，新改扩建城市道路60公里。新建、提升木兰溪沿岸景观带、城港大道等绿道30公里，推进绿心、绶溪、玉湖、植物园等公园建设，新增公园绿地面积50公顷以上。加快城市公共自行车系统建设，新建人行天桥8座，拓展公共活动空间，完善社区综合服务设施，新增停车泊位3000个以上，让群众生活更加舒适便利。

　　提高城市管理服务水平。加快建设智慧城市公共信息平台，完善城市管理基础数据库，大力解决市政维护、物业管理、停车难、“两违”整治等问题，提高城市精细化管理水平。开展街道设计，推进城市家具、广告招牌等设施标准化建设，着力开展人行道净化，继续推进生活垃圾分类，提高城市规范化管理水平。加强供排水、供气、道路桥梁等市政公用设施风险排査和隐患治理，提高城市安全管理水平。

　　推进人文城市建设。弘扬社会主义核心价值观，巩固提升全国文明城市创建成果，深化群众性精神文明创建，大力发展志愿者服务组织。传承保护莆仙戏、十音八乐、传统技艺等莆仙文化。加强宋城、萝苜田历史文化街区和古村落保护，创建国家历史文化名城。提升“四馆一宫”服务水平，推广莆阳书房模式，加强基层综合性公共文化服务中心建设，丰富文化产品和服务供给。培育一批龙头骨干文化企业。完善公共体育服务，推动竞技体育发展，办好2019年国际射联步手枪世界杯总决赛、第三届“妈祖杯”海上丝绸之路国际羽毛球挑战赛等赛事，发展体育产业。

　　（五）大力实施乡村振兴战略，加快推进农业和农村现代化进程，开创新时代“三农”发展新局面

　　打好精准脱贫攻坚战。深入实施脱贫攻坚三年行动计划，坚持把提高脱贫质量摆在首位，健全贫困人口动态管理和稳定脱贫长效机制，精准落实产业、就业、健康、低保兜底保障等扶贫措施，实现88个贫困村脱贫摘帽全覆盖，确保政策落实一个不漏、小康路上一个不少。实施贫困村提升行动，每个贫困村发展1个以上重点产业扶贫项目。推进村级集体经济“破壳消薄”，实现全市村级集体年经营性收入超10万元。动员社会力量积极参与脱贫工程，构建大扶贫工作格局。

　　实施特色现代农业工程。切实保障粮食安全，加快发展特色农业、生态农业和精品渔业，新增20家市级以上农业产业化龙头企业，新建20个优质农产品标准化生产示范基地，建成20个农产品初加工中心，培育10个以上“一村一品”示范村。巩固提升现代渔业，发展远洋捕捞，推进南日岛海洋牧场示范区建设，建设“蓝色粮仓”。依托振兴乡村平台，举办优质农产品文化节，打响“莆田海鲜”“莆田桂圆”等公共品牌。

　　优化农村人居环境。实施农村人居环境整治三年行动、乡村振兴“百村示范”工程，开展铁路沿线环境治理，提升村庄基础设施和公共服务水平，建设一批连线成片的特色景观带，打造美丽宜居乡村。开展“一革命四行动”，新建改造乡村公厕81座；加强村庄规划管理，引导集中建设村镇住宅小区，70%以上的村庄完成房前屋后整治；推进村庄“四旁”绿化，提升田园风光品质；强化农村垃圾治理，实现日常保洁无死角；新建改造6.97万户三格化粪池，建设6个乡镇污水处理设施，实现乡镇生活污水处理设施全覆盖。提升“四好农村路”创建水平，改善农村出行条件。

　　健全城乡融合发展机制。深化新型城镇化和城乡一体化试点，整合资源，完善配套，打造出一批就地城镇化、就近市民化的样板。加快荔城区、仙游县农村集体产权制度改革试点，推进农村经营性资产股份合作制改革，促进农村要素和城市资本、人才、技术的有效对接，推动资源要素更多流向农村。深入推行科技特派员制度，支持各路人才“上山下乡进岛”、投身乡村振兴。

　　（六）坚定新时代改革开放再出发的信心决心，在更高层次推动莆田新一轮改革开放，激发经济社会发展活力

　　全方位支持民营经济。牢记“民营企业和民营企业家是我们自己人”，全面构建有温度、能感知的亲清政商关系。深入开展民营企业大走访大调研大服务活动，进企业、送政策、解难题，坚决落实好减负降费各项政策，推动涉企行政事业性收费实现“零收费”，设立纾困基金，发挥中小企业融资担保资金作用，搭建“金融便利超市”，加大对民营企业融资的扶持和引导，破解企业融资难题，创新“网购式”服务模式，建立服务企业联席会议、知识产权专家库、企业首席服务官制度，完善企业家意见受理、处置、反馈的常态化机制，解决好企业反映的环保、用工、办证等问题，真正营造“懂民企、爱民企、帮民企”的良好氛围，让民营企业安安心心谋发展、心无旁骛干实业。

　　推进重点领域和关键环节改革。全面深化、系统总结各项国家级、省级改革试点，创造更多有影响、可复制的改革样板。打好防范化解重大风险攻坚战，妥善化解隐性存量债务，坚决防控金融风险，严厉打击非法集资和恶意逃废债行为，金融机构不良率控制在2%以内。加快国有企业实体化改革，以市场化为导向，加快资产盘活和专业团队建设，建立健全现代企业管理制度，提升经营运作水平。深化医保支付方式改革，建立医疗服务价格和医保支付标准体系。完成新一轮政府机构改革。完成第四次全国经济普查任务。

　　发展更加高效的开放型经济。实施“助力千企外贸成长”计划，大力支持企业参与国际性展会，建立境外销售网络和中转基地，开拓多元化国际市场。发展信息服务、研发设计、文化创意等新兴服务贸易，举办第二届“一带一路”跨境电商国际合作高峰论坛，扶持一批“买全球、卖全球”的跨境电商龙头企业，促进贸易提质增效。强化“一把手”招商，赴境外洽谈对接一批大项目好项目，实现利用外资增量与质量双提高。深化对台交流交往，加大莆台医疗健康、电子信息、精密机械等产业对接，建设两岸健康产业交流合作基地。推进莆港、莆澳新一轮交流合作，鼓励海外侨胞回乡投资兴业。

　　发挥港口对外开放带动作用。以莆田港东吴港区扩大开放获批为契机，加快东吴港区、秀屿港区连片开发，建设东吴港区3-6#临港产业码头和集装箱运输场站，推进装配式建筑产业基地等一批临港工业项目建设。积极发展保税和转口贸易，开展罗屿作业区进口铁矿石保税混（配）矿业务，构建东南沿海铁矿石中转基地。大力发展水水中转和海陆联运，支持船代、货代等航运服务企业升级做大。全面实行口岸收费目录清单制度，深化物联网、云计算等信息技术应用，提升口岸跨境贸易便利化水平，放大港口对外开放的综合效应。

　　培植区域协同发展新优势。推进基础设施互联互通，加快建设福厦客专、联十一线等协作项目，开工建设福莆宁城际铁路F2线一期工程，争取开通对台直航。促进产业协同配套，大力推动华佳彩、佳通轮胎、云度汽车等企业与闽东北协同发展区内企业互为采购，积极参与振华重工集团的战略合作。强化公共服务共享，推动医疗资源和卫生应急联动协作，开展医院管理、技术指导、人才培养、双向转诊、远程会诊等全方位医疗卫生合作。

　　（七）践行习近平总书记治理木兰溪的重要理念，全面提升生态环境质量，打造人与自然和谐共生的莆田样板

　　推进木兰溪全流域系统治理。围绕构建水安全、水资源、水环境、水生态、水经济、水文化等“六大体系”，推进木兰溪流域沿线18个乡镇污水收集处理，全面整治入河排污口，系统整治南北洋水系，提升木兰溪全流域防洪排涝能力。实施沿溪两岸城市景观工程，建设木兰陂至玉湖片区生态景观带。抓好木兰溪流域的生态修复、分类管控、湖泊提升工作，启动生态绿心的规划保护利用工作，确保木兰溪流域水质优良比例达83.3%以上。

　　坚决打好污染防治攻坚战。实施打赢蓝天保卫战三年作战计划，全面整治“散乱污”企业，推广挥发性有机物治理标杆模式，力争空气质量优良天数达95%，让蓝天白云、繁星闪烁成为常态。深入落实“河长制”，创新河道整治及运营机制，确保省控重点小流域水质优良比例达82.7%以上，创建国家城市黑臭水体治理示范城市。全面实施土壤污染防治行动计划，强化固废规范化管理，推进土壤污染状况调查及修复试点。持续抓好中央、省环保督察反馈问题整改。

　　加强生态保护修复。强化生态红线管控，合理控制空间开发强度。落实《莆田市东圳库区水环境保护条例》，深入实施城乡供水水质提升三年行动，加快城乡供水一体化试点，县级及以上集中式饮用水源地水质达标率稳定在100%。加强自然保护地管理，开展“绿盾”自然保护区监督检查专项行动，完成“三区两线”内53个废弃矿山生态环境修复治理。加强陆海污染源监管和海洋环境监测，严查各类海洋违法行为。

　　健全生态环保长效机制。支持开展生态环保公益诉讼，落实生态环境保护责任追究制度和环境损害赔偿制度，严格环境监管和执法。落实企业环境主体责任，建立绿色信贷贴息机制，创新排污权抵质押贷款，开展企业环境信用评价，在环境高风险领域建立环境污染强制责任保险制度。开展全民绿色行动，倡导绿色生活方式，创新多元主体共建共治共享机制。

　　（八）坚持以人民为中心的发展思想，加快补齐民生短板，不断增进民生福祉

　　深化教育强市建设。落实《莆田市中小学校幼儿园规划建设条例》，坚持公办民办并举，通过购买服务、综合奖补、派驻公办教师等方式，支持普惠性民办幼儿园发展，扩大普惠性学前教育资源供给，新增普惠性幼儿学位1万个。实施中小学改扩建工程，新增中小学学位7000个。规范校外培训机构。实施教育“三名”工程，加快教育集团和教育联盟建设。全面深化新时代教师队伍建设改革，完善“县管校聘”机制，逐步提高教师待遇，进一步激发教师队伍活力。加快东南新工科产业学院、莆田学院新校区建设，实现湄职院主体搬迁，开工建设东南医学院。深化产教融合、校企合作，构建全民终身教育平台，推行终身职业技能培训，提高劳动者素质和就业创业能力。

　　推进健康莆田建设。继续实施健康莆田行动规划，推动从治疾病向促健康转变。加强健康服务，实施健康“三名”工程，建成投用国药涵江医院、城厢区医院、爱丁堡国际医院、口腔医院、瑞士国际康复医院，开工建设瑞斯自闭症治疗中心，全市新增病床位1700张以上、千人均病床位6张以上。加强健康管理，推进疫苗流通使用专项治理，大力发展智慧医疗、普惠医疗，健全居民电子健康档案，做实家庭医生服务。倡导健康生活，普及健康知识，提高公共卫生服务水平。创建国家食品安全示范城市，保障群众“舌尖上的安全”。

　　健全社会保障体系。实施就业创业“十百千”计划，推动高校毕业生、就业困难人员等重点群体实现更充分就业，新增城镇就业1.7万人。深入实施全民参保计划，提高城乡居民养老保险政府代缴和补贴标准，常住人口基本养老保险参保率达95%。推进医养结合，引导更多社会资本进入养老服务业，开展居家养老应急服务，建设农村幸福院100个、社区日间照料中心10个。着力解决被拆迁群众回迁难、办证难问题，全面开展安置回迁办证清理活动。推行土地出让配建安置房回购方式，提升安置工作水平。加大社会救助力度，发展残疾人事业，关爱留守儿童、空巢老人和务工人员随迁子女。

　　构建有效社会治理体系。推进平安莆田全域创建，推动社会治理重心向基层下移。深化扫黑除恶专项斗争，加快社会治安防控体系建设，依法打击和惩治违法犯罪活动。加强社会风险评估和矛盾纠纷排查化解，推进信访规范化、信息化、法治化建设，构建大调解工作格局。持续开展安全生产专项整治，提升应急处置能力，推进城市安全发展。落实军民深度融合发展战略，加强双拥优抚安置工作。支持工会、工商联、共青团、妇联等群团组织更好发挥作用。

　　三、全面深化服务型政府建设

　　各位代表！新时代，我们肩负新使命，要有新担当新作为。我们将深入贯彻习近平新时代中国特色社会主义思想，树牢 “四个意识”， 坚定 “四个自信”，坚决做到“两个维护”，牢记党的宗旨，加强自身建设，推动各项工作再创新业绩。

　　牢固树立法治理念，深化法治政府建设。全面落实机构改革总体要求，切实提升政府依法行政水平和履职能力。自觉接受人大法律监督、工作监督，主动接受政协民主监督，认真办理人大代表建议和政协提案，充分听取各民主党派、工商联、无党派人士和各人民团体的意见，接受监察和审计监督。严格落实重大行政决策合法性审查制度，完善权力、责任、监管清单，加强政府行政规章及规范性文件制定和监督管理，推进重大行政决策科学化、民主化、法治化。强化行政复议和行政应诉，深化政务公开、府院联动和“两法衔接”机制，深入推进“七五”普法。

　　弘扬改革创新精神，持续优化营商环境。加强干部知识更新，强化干部能力提升，坚持正向激励和容错纠错机制，鼓励探路先试，包容改革失误，激发干部干事创业热情。聚焦企业关切，建立政府服务企业大家评制度，深化营商环境第三方评价机制及结果应用。深化“放管服”改革，巩固“多证合一”成果，简化企业开办程序，推进工程建设项目审批制度全流程全覆盖改革，推广投资项目承诺制，让放权更彻底、更到位。全面推行“双随机一公开”，运用“互联网+监管”新模式，让监管更科学、更高效。深入推进审批服务便民化和“减证便民”行动，加快实现“只进一门，一窗受理，只跑一次”，让办事更快捷、服务更精准。

　　秉持求真务实作风，狠抓各项工作落实。坚决破除不符合新时代发展要求的各种思想和观念，创造性贯彻落实上级各项部署，大力弘扬“马上就办、真抓实干”作风，瞄准目标、坚定信心，一张蓝图绘到底，不断完善政策执行方式，提升政府执行力。始终保持同人民群众的血肉联系，从群众最关心、最需要的事情做起，开展群众办事百项堵点疏解行动，财政支出优先投向民生事业，办好25件市委市政府为民办实事项目。大兴调查研究之风，精简检查考评事项，实现检查考核总量减少50%以上，减轻部门和基层负担，让大家从无谓的事务中解脱出来。

　　坚定抓好从严治政，加强廉洁政府建设。坚持管党治党责任与政府工作责任一起落实，深入推进“两学一做”学习教育常态化制度化，开展“不忘初心，牢记使命”主题教育，切实把全面从严治党“八个坚定不移”要求落到政府工作全过程。强化意识形态工作责任制，严守政治纪律和政治规矩。完善工程招投标、扶贫惠农、教育医疗等领域廉政风险防控制度，坚决整治侵害群众利益的不正之风和腐败问题。从严控制“三公”经费支出，提高财政资金使用效益。

　　各位代表，时代赋予重任，拼搏铸就辉煌。让我们更加紧密团结在以习近平同志为核心的党中央周围，在省委省政府和市委的坚强领导下，以只争朝夕的劲头、坚韧不拔的毅力，奋力奔跑、追逐梦想，以建设美丽莆田的优异成绩，落实高质量赶超任务，庆祝新中国成立70周年！
